# Successful radiofrequency catheter ablation of right-sided focal atrial tachycardia in a patient with Holt–Oram syndrome: a case report

**DOI:** 10.3389/fcvm.2026.1791250

**Published:** 2026-08-18

**Authors:** Dejan Kojić, Anja Radunović, Sladjana Božović-Ogarević, Ivan Ilić, Vuk Niković, Milosav Tomović, Milovan Bojić

**Affiliations:** 1 Cardiology Clinic, Department of Electrophysiology and Electrostimulation, Institute for Cardiovascular Diseases Dedinje, Belgrade, Serbia; 2 Faculty of Medicine, University of Belgrade, Belgrade, Serbia; 3 Emergency Medical Center of Montenegro, Podgorica, Montenegro

**Keywords:** ASD closure, atrial septal defect (ASD), atrial tachycardia (AT), Holt–Oram syndrome (HOS), radiofrequency catheter ablation

## Abstract

**Background:**

Holt–Oram syndrome (HOS) is a rare autosomal-dominant genetic disorder caused by mutations in the TBX5 gene. It is characterized by skeletal abnormalities of the upper limbs and congenital heart defects, most frequently atrial septal defect (ASD) and ventricular septal defect. Middle-aged individuals often develop conduction disorders and atrial arrhythmias, most commonly atrial fibrillation and atrial flutter, while focal atrial tachycardia (AT) remains relatively rare. We present the successful catheter ablation of a right anteroseptal focal AT in a patient with HOS and a history of surgical ASD closure.

**Case report:**

A 22-year-old man was admitted due to recurrent episodes of supraventricular tachycardia, refractory to drug therapy. He has a history of HOS, patch closure of ASD at age three, and three hand surgeries. A 12-lead electrocardiogram showed narrow QRS complex tachycardia at 171 beats/min, regular RR intervals, and RP < PR interval. Transthoracic echocardiography revealed mostly normal findings, with mildly reduced left ventricle function and intact ASD patch with no residual shunt. Electrophysiology study showed no retrograde conduction over an accessory pathway. Programmed stimulation from the left atrium induced tachycardia with cycle length 280 ms, narrow QRS complexes, regular RR intervals, initial 1:1 AV (atrio-ventricular) ratio, and short VA interval (34 ms). Using the Carto 3 3D mapping system, activation mapping of the right atrium identified the earliest atrial activation near the His-Bundle area (5.9 mm distance from His bundle cloud). Radiofrequency ablation terminated AT within 2.5 s of the first application. Postablation tachycardia was not-inducible at baseline and under Isoproterenol. The patient was discharged from the hospital 1 day after ablation without antiarrhythmic therapy. After 21 months of follow-up, there were no recurrences of AT.

**Conclusion:**

This case demonstrates that catheter ablation is a feasible, safe, and effective treatment option for focal AT patients with HOS and surgical ASD closure.

## 1 Introduction

Holt–Oram syndrome (HOS), also known as heart–hand syndrome, was first described in 1960 by Mary Holt and Samuel Oram (1–3). It is a rare autosomal-dominant genetic disorder with an estimated prevalence of 1 in 100,000 live births in the USA (2). Since the discovery of TBX5 (T-Box Transcription Factor 5) in 1997, different variants have been reportedly associated with both familial and sporadic cases of HOS. TBX5 is a transcription factor and a member of the T-box family essential for cardiac development, particularly development of the conduction system, septation, and cardiomyogenesis (4).

Clinically, HOS is characterized by skeletal abnormalities of the upper limbs and congenital heart defects, most frequently atrial septal defect (ASD)—particularly type secundum—ventricular septal defect, most commonly in the muscular trabecular septum (5, 6). Approximately 85%–95% of patients with HOS present with some form of cardiac abnormality. (6) In addition to structural defects, conduction system disease and atrial arrhythmias (AAs) are common and tend to progress with age (7). Middle-aged individuals frequently develop conduction disorders and AAs, including atrial fibrillation (AF) and atrial flutter (AFL), whereas focal atrial tachycardia (AT) is relatively rare (8).

Left-to-right shunting associated with ASD results in cardiac geometrical and electrical remodeling of both atria, and changes in atrial tissue properties. These changes— including interstitial fibrosis, myocyte size enlargement, and ultrastructural changes—increase the incidence of AAs, such as AF and AFL (8, 9). Previous studies have reported inconsistent findings on the impact of ASD closure on atrial arrhythmia burden, particularly in patients over 40 years of age, with some reports indicating a decrease and others showing no meaningful change in overall burden (10–13).

In long-term follow-up after ASD closure, the prevalence of AAs gradually increases, reaching approximately 4.9% by 6 years postclosure, with higher rates in older patients (>60 years of age). The most frequent AA during the postclosure period is AFL followed by AF. Focal AT is extremely rare (14).

Atriotomy scar and presence of atrial patch in patients with surgical closure of ASD, and device-related inflammation and scarring in patients underwent interventional closure of ASD, can be part of arrhythmia circuits (8, 9, 15). Although antiarrhythmic drugs are often used to manage AAs, their long-term efficacy is limited (16).

Catheter ablation (CA) of AF, AFL, or focal AT is a safe and effective treatment option, with reported success rates 80%–95% and low complication rates, making catheter ablation a feasible modality for long-term management (16, 17).

In this case report, we present the successful CA of a right-sided anteroseptal focal AT in a patient with HOS and a history of surgical ASD closure.

## 2 Case report

A 22-year-old man was admitted to our institution for electrophysiology study and catheter ablation of supraventricular tachycardia. Over the past few years, he had experienced recurrent episodes of supraventricular tachycardia. Treatment with beta blockers and calcium channel blockers was not effective. He still had symptomatic episodes of tachycardia twice a week, frequently requiring medical intervention or hospitalization.

**Figure 1:**
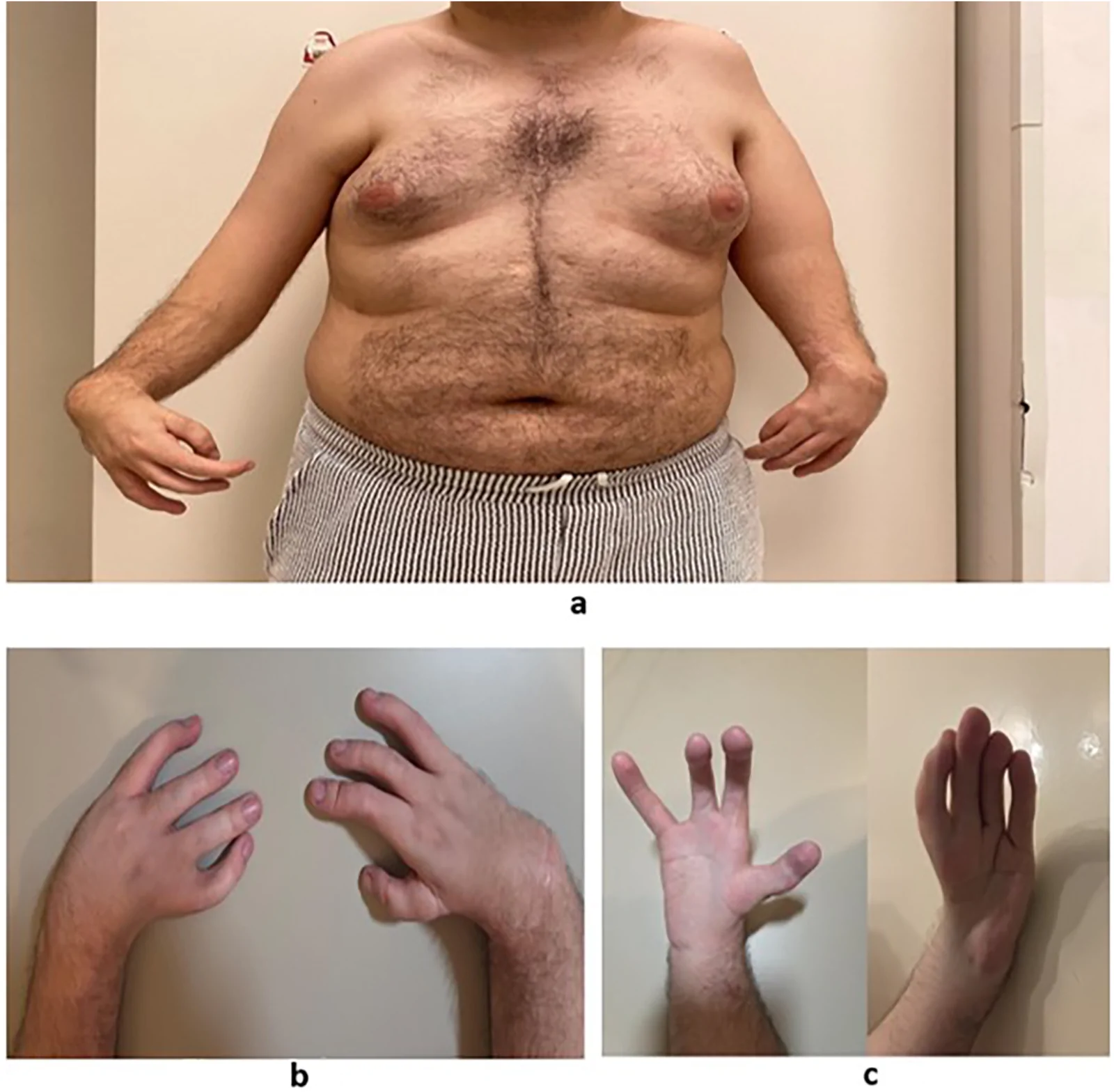
**(a)** Typical upper-limb deformities in Holt–Oram syndrome, and **(b,c)** hand deformities in Holt–Oram syndrome.

He had a history of HOS with typical upper-limb defects (Figures 1a–c) and ASD. Surgical patch closure of ASD was performed at the age of three. He also underwent three hand surgeries due to congenital radial aplasia during infancy and childhood.

**Figure 2:**
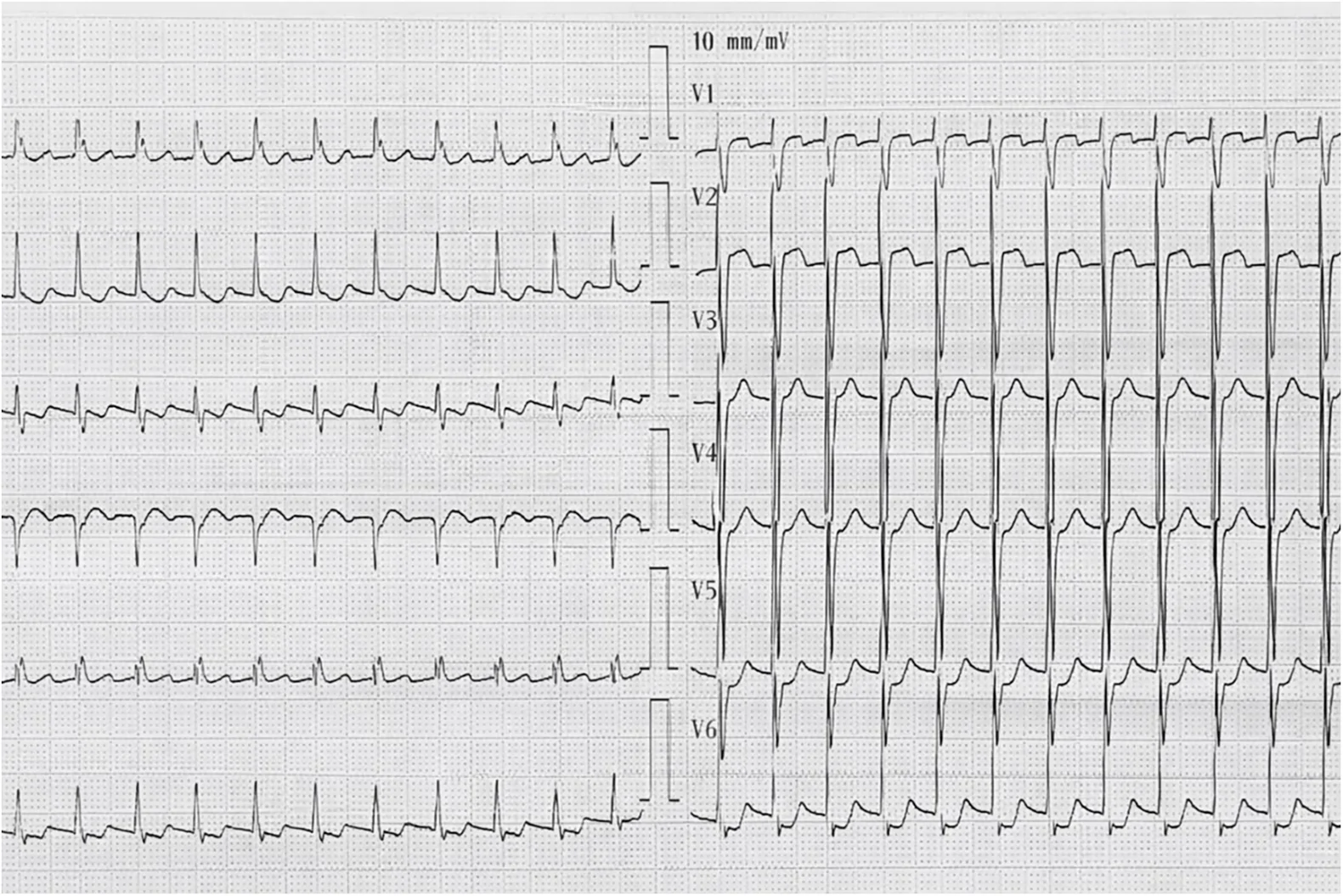
A 12-lead ECG demonstrating regular narrow-QRS complex tachycardia at a heart rate 171 per minute, with a short RP interval (RP < PR interval).

A 12-lead electrocardiogram (ECG) during tachycardia (Figure 2) showed narrow QRS complex tachycardia with a heart rate of 171 per minute, regular RR intervals, and RP < PR interval. There was no manifest ventricular pre-excitation during sinus rhythm and no conduction abnormalities. Transthoracic echocardiography showed normal dimensions of the left ventricle (LV) with mildly reduced LV ejection fraction and no regional wall-motion abnormalities. The left atrial diameter was normal. The right ventricular (RV) diameter was normal, with preserved RV systolic function. There were no signs of pathological flow across the interatrial septum. Valves were competent.

**Figure 3:**
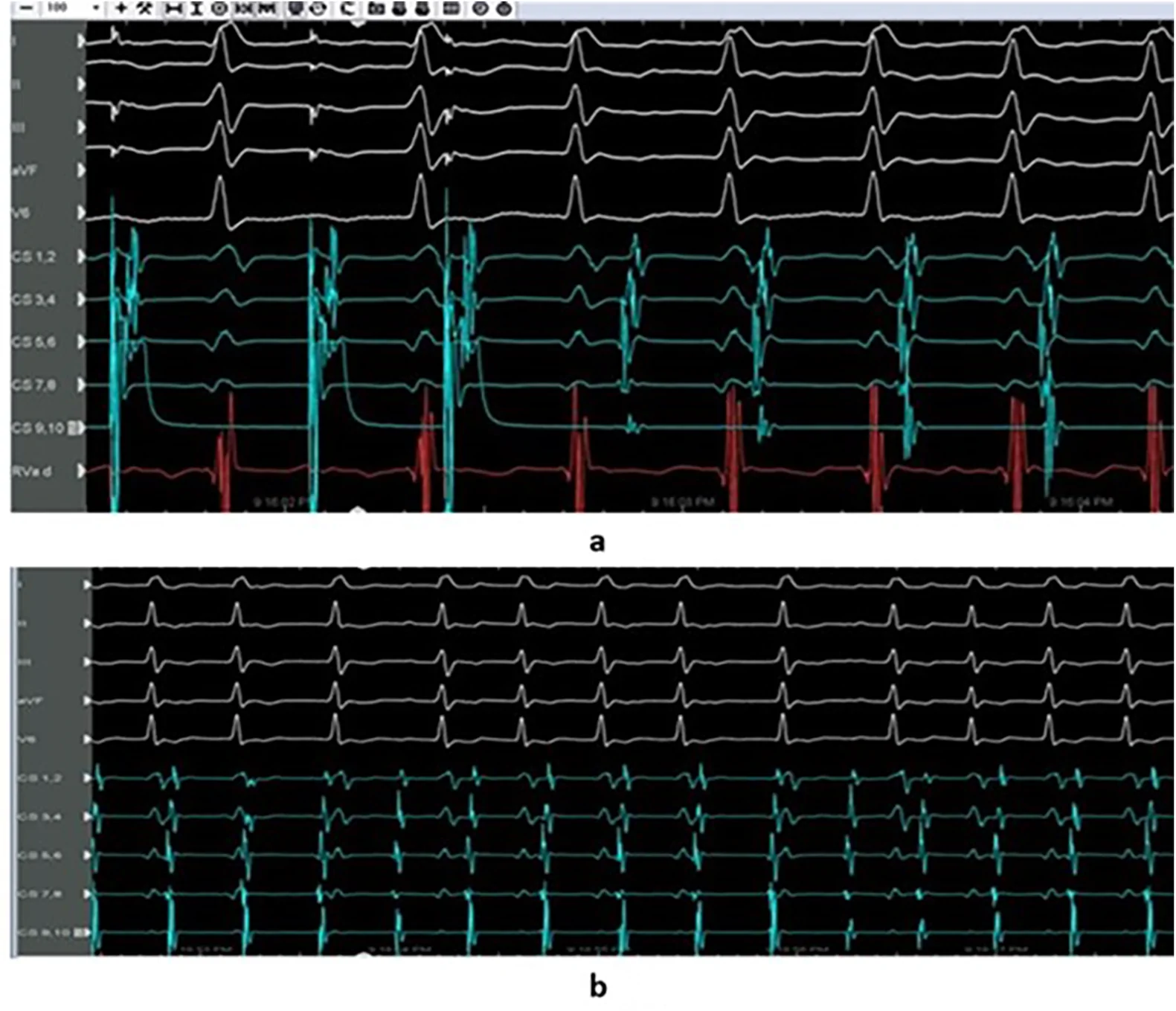
**(a)** Induction of regular narrow-QRS tachycardia (CL 280 ms) by programmed stimulation from left atrium, with 1:1 AV relationship and a short VA interval (34 ms), and **(b)** irregular narrow-QRS complex tachycardia with AV dissociation, atrial rate 170/min, faster than ventricular rate (A > V), consistent with atrial tachycardia and variable AV conduction.

The patient underwent electrophysiology study. Through the right femoral vein, we placed catheters in the following positions: coronary sinus (CS, proximal bipole 9–10 at CS ostium), His bundle (HB), and right ventricle apex (RV). Baseline intracardiac intervals were normal. During retrograde pacing from the RV apex, we documented complete VA block, excluding retrograde conduction over an accessory pathway. Using programmed stimulation with extrastimuli from the left atrium, we induced tachycardia with cycle length (CL) 280 ms, narrow QRS complexes, regular RR intervals, initial 1:1 AV ratio, and short VA interval (34 ms) (Figure 3a).

**Figure 4:**
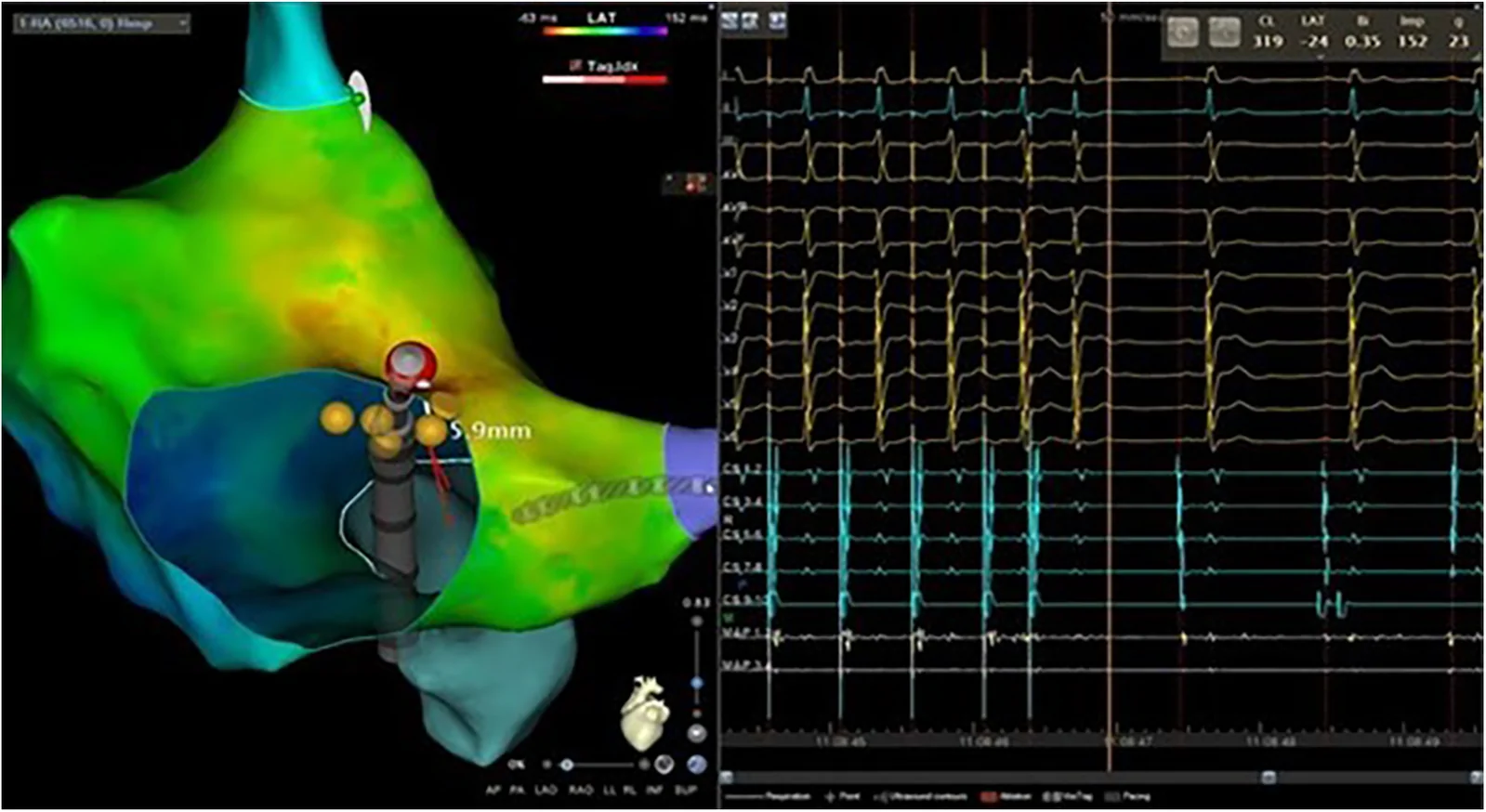
Left panel: activation map of the right atrium in left anterior oblique view, showing the site of successful radiofrequency ablation (red dot) in proximity to HB area, 5.9 mm from HB cloud (yellow dots). Right panel: Termination of AT after 2.5 s of the first radiofrequency energy application.

Subsequently, the tachycardia spontaneously became irregular with AV dissociation, and faster A than V, consistent with AT (Figure 3b). Right ventricular overdrive pacing with V-A-A-V response upon cessation confirmed the diagnosis of AT. We used the Carto 3 3D electroanatomical mapping system (Biosense Webster, Diamond Bar, CA, USA) and a Pentaray mapping catheter (Biosense Webster, Diamond Bar, CA, USA) to create an activation map of the right atrium during tachycardia. The activation map showed the earliest atrial activation located in close proximity to the HB area (5.9 mm from the HB cloud), consistent with focal right-sided anteroseptal (para-Hisian) atrial tachycardia (Figure 4). The earliest local activation time was −19 ms.

Radiofrequency (RF) ablation was performed using a NaviStar Thermocool SmartTouch catheter (Biosence Webster), with a power setting of 30 W, a temperature limit of 43°C, and saline irrigation at a flow rate of 17 mL/min. Tachycardia was terminated within 2.5 s of power delivery during the first application of RF energy at the site of earliest activation (Figure 4, right panel). Throughout the ablation procedure, AV conduction was carefully monitored by continuous assessment of the PR interval. No PR prolongation or other evidence of AV nodal conduction impairment was observed during energy delivery. AT the end of the procedure, tachycardia was not inducible under basic conditions and after facilitation with isoproterenol. The patient was discharged from the hospital the day after the ablation without antiarrhythmic therapy. At 21-month follow–up, there were no recurrences of AT.

## 3 Discussion

To the best of our knowledge, this is the first report of successful catheter ablation of focal AT in a patient with HOS and history of ASD closure. HOS is caused by mutations in the TBX5 gene, which directly regulates several genes involved in cardiac electrical activity, such as GJA5 (Connexin40) and SCN5A, which are essential for cardiac excitability and propagation of impulses (18, 19). The most commonly reported arrhythmias in HOS include sinus node dysfunction, first-degree to complete AV block, and AF/AFL (6, 20). Some mutations, particularly missense mutations like TBX5 p.Gly125Arg, have been associated with gain-of-function effects, enhancing transcription of conduction-related genes and promoting arrhythmic phenotypes. A murine model carrying this mutation replicated several hallmark features of human arrhythmogenic HOS, including spontaneous atrial ectopy, prolonged action potentials, and AV block (19). Clinical manifestations of HOS are highly variable, often requiring a multidisciplinary approach.

ASD is one of the most common congenital heart defects and can be associated with AAs, right-sided heart failure, and pulmonary hypertension. Patients with ASD have a significantly higher risk of developing AAs compared with the general population due to progressive structural and electrical remodeling of the atrial myocardium. The mechanism of arrhythmogenesis in ASD includes chronic volume overload of the right atrium, leading to dilatation, fibrosis, and altered atrial conduction. Even after ASD closure, particularly in older patients, arrhythmic risk persists or increases due to incomplete reverse remodeling (8, 9).

A meta-analysis by Vecht et al. demonstrated that while ASD closure reduces the prevalence of AAs in the short to medium term, it does not consistently prevent late arrhythmias (10). A significantly greater risk is particularly notable in patients with larger right-sided chambers, elevated or higher pulmonary pressures, or preexisting AAs (21). Furthermore, while geometric remodeling may improve postclosure, electrical remodeling is less reversible, often leaving a substrate for arrhythmia circuits (8). In a retrospective study of 517 patients after transcatheter closure of ASD, Chiu et al. identified that age, size of the occluder, preimplantation atrial arrhythmia, metabolic syndrome, thyroid dysfunction, and mitral valve disease were significant risk factors for late AF/AFL development (22).

Burke et al. reported that the prevalence of AAs demonstrated a biphasic pattern following ASD closure, with an early peak of approximately 14% occurring about 1 week after the procedure, followed by a reduction to 3.4% at 6 months during follow-up. In the long term, the prevalence gradually increased, reaching approximately 4.9% at 6 years postclosure. The most frequent AA during the postclosure period was AFL followed by AF. Focal AT was extremely rare (14).

## 4 Conclusion

This case demonstrates that radiofrequency catheter ablation can be a safe and effective treatment for focal atrial tachycardia in patients with Holt–Oram syndrome and prior surgical ASD closure. Although atrial arrhythmias are well recognized in these patients, focal atrial tachycardia appears to be rare, particularly decades after ASD repair. The present case provides additional clinical evidence to support effective arrhythmia management in this uncommon genetic condition.

The successful ablation of a right-sided para-Hisian focal AT without complications highlights the feasibility of catheter ablation in this challenging anatomical setting when guided by detailed three-dimensional electroanatomical mapping.

## 5 Limitations

Several limitations should be acknowledged. A comprehensive substrate characterization was not performed in this case. Although activation and voltage mapping enabled identification of the earliest activation site, where ablation consistently terminated the tachycardia, extensive mapping was avoided because of the anteroseptal location and the potential risk associated with mapping close to the AV node in a surgically modified atrium. Therefore, successful ablation at a single earliest activation site cannot be considered definitive evidence of focality or substrate simplicity. Rather, the electrophysiological findings should be interpreted in the context of a pragmatic, safety-oriented procedural strategy in an exceptionally rare and anatomically complex clinical setting.

## Data Availability

The raw data supporting the conclusions of this article will be made available by the authors, without undue reservation.
